# Supplementary material for: IL-21/IL-21R Promotes the Pro-Inflammatory Effects of Macrophages during C. muridarum Respiratory Infection
Source: Int J Mol Sci. 2023 Aug 8;24(16):12557. doi: 10.3390/ijms241612557 (PMC10454239; doi:10.3390/ijms241612557)
Supplement: Supplementary file 1 [file ijms-24-12557-s001.zip › ijms-2528197-supplementary.pdf]

IL-21/IL-21R Promotes the Pro-Inflammatory Effects of Macrophages during *C. muridarum* Respiratory Infection

Supplementary figures:

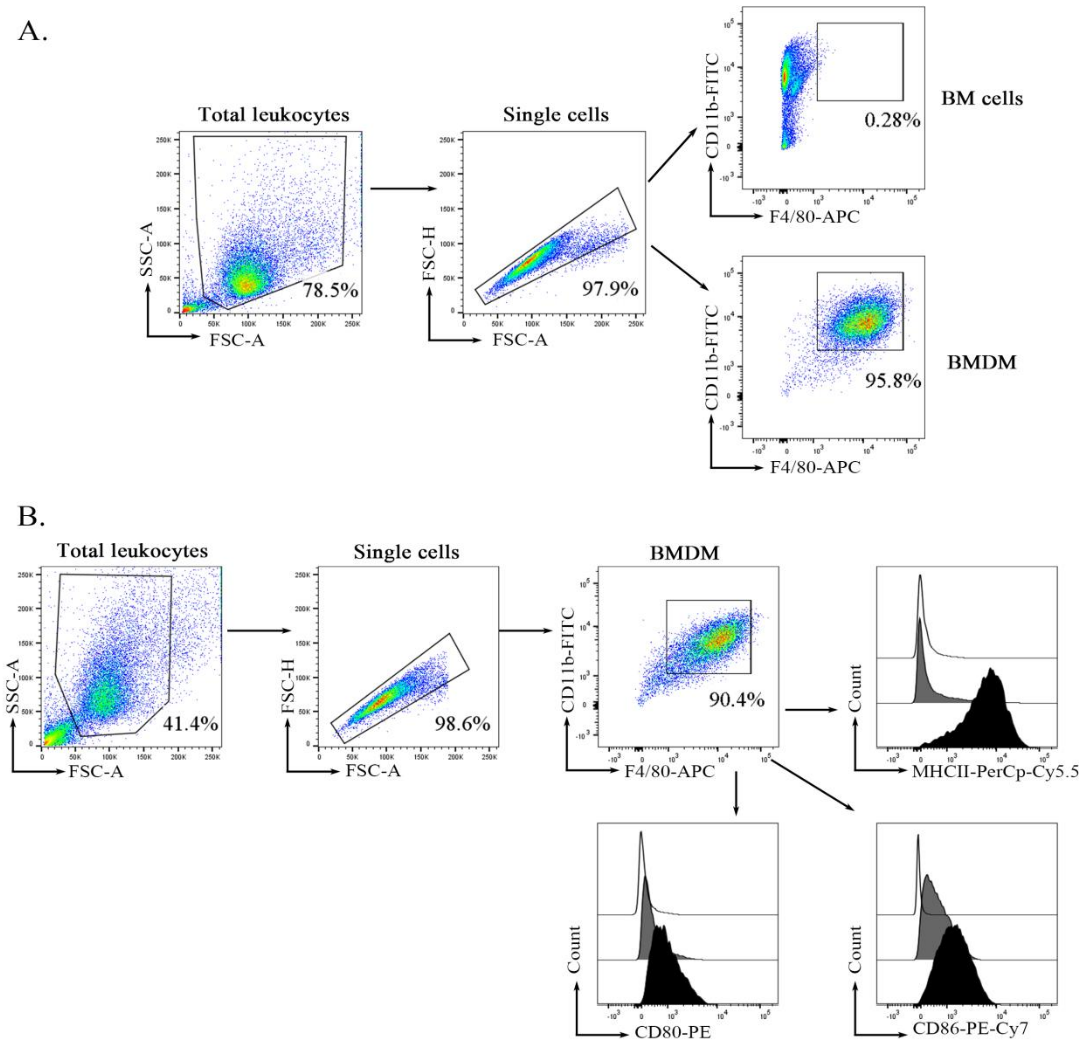

Supplementary Figure S1. Gating strategy for determination that bone marrow (BM) cells were induced into bone marrow-derived macrophages (BMDM) and M1-like BMDM. Tibia and femur of naive WT and IL-21R$^{-/-}$ mice were isolated, and bone marrow was extracted to induce macrophages and M1-like macrophages in vitro. Cells were harvested at the designated time point and subjected to flow cytometric analysis. (A) Representative flow cytometric images of BM cells and BMDM cells. Debris and doublets were first excluded from the single cells. The F4/80^{+}CD11b^{+} cells were identified as BMDM. (B) Flow cytometry was performed on in vitro polarized M1-like BMDMs. Following the exclusion of debris and doublets from the single cells, we proceeded to perform gating for MHC II^{+}, CD80^{+} and CD86^{+} cells on F4/80^{+}CD11b^{+} BMDMs. (White, FMO; Grey, BMDM; Blank, BMDM-M1)

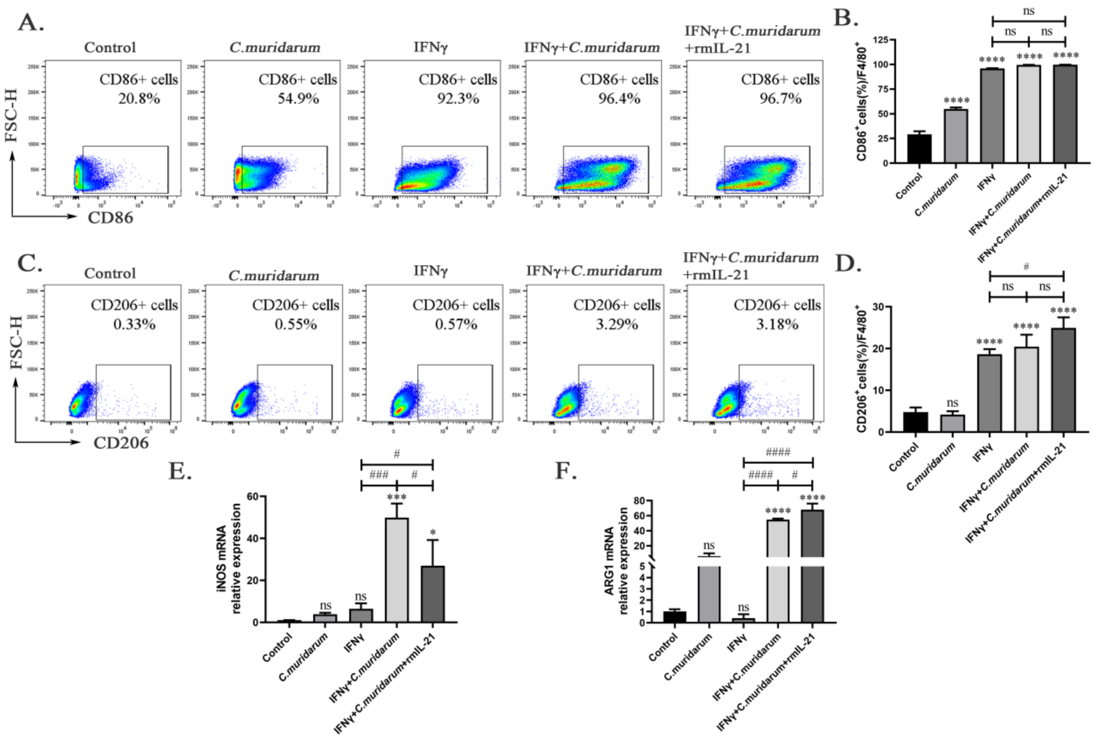

Supplementary Figure S2: IL-21 promoted ARG1 mRNA expression and suppressed iNOS mRNA expression in *C. muridarum*-stimulated M1-like macrophages in vitro. RAW264.7 cells were stimulated with IFNγ (100ng/ml, a cytokine inducing M1 marker) for 24 hours to polarize them into M1-like macrophages. This was followed by the addition of *C.muridarum* and/or rmIL-21 for another 24 h. (A and C) Representative flow cytometric images of CD86^{+} cells (A) and CD206^{+} cells (C) within F4/80^{+} cells derived from stimulated RAW264.7 cells. (B and D) Flow cytometry data are summarized to show the percentages of CD86^{+} cells (B) and CD206^{+} cells (D) within F4/80^{+} cells derived from stimulated RAW264.7 cells. (E and F) The mRNA expressions of iNOS (E) and ARG1 (F) were analyzed via qPCR. Data are presented as means ± SD from three multiple wells for each group, representative of one of three independent experiments. Statistical significance of differences was determined by one-way ANOVA. * $p < 0.05$, *** $p < 0.001$, ****$p < 0.0001$, # $p < 0.05$, ### $p < 0.001$, #### $p < 0.0001$. "*" indicates a comparison within a group or with the Control group. "#" refers to a comparison between the IFNγ and IFNγ+*C. muridarum* groups, the IFNγ and IFNγ+*C.muridarum*+ rmIL-21 groups or between the IFNγ+*C. muridarum* and IFNγ+*C.muridarum*+ rmIL-21 groups. "ns" indicates not significant.
